# The complete chloroplast genome of *Hippeastrum rutilum* (Ker-Gawl.) Herb.

**DOI:** 10.1080/23802359.2021.1899068

**Published:** 2021-03-24

**Authors:** Li He, Bowen Zou, Haidong Gao, Lei Liu, Yang Wu

**Affiliations:** a College of Life Science, Jinggangshan University, Ji’an, Jiangxi, China; b Genepioneer Biotechnologies Co. Ltd, Nanjing, China

**Keywords:** Amaryllidaceae, *Hippeastrum rutilum* (Ker-Gawl.) Herb, chloroplast genome, phylogenetic

## Abstract

*Hippeastrum rutilum* (Ker-Gawl.) Herb. is a high-end ornamental flower in the family Amaryllidaceae. In this study, the complete chloroplast genome sequence of *H. rutilum* (Ker-Gawl.) Herb. was determined from Illumina pair-end sequencing data. The sequencing results indicated the complete chloroplast genome sequence of *H. rutilum* (Ker-Gawl.) Herb. 162,696 base pairs (bp) in length, including one large single-copy region (LSC, 86,933 bp), one small single-copy region (SSC, 5418 bp), and a pair of inverted repeat regions (IRs) of 34,932 bp. Besides, the complete chloroplast genome contained 128 genes, including 82 protein-coding genes, 38 tRNA, and 8 rRNA. The phylogenetic analysis suggested that *H. rutilum* (Ker-Gawl.) Herb. is closely related to *H*. *rutilum*. The complete chloroplast genome sequencing results will provide a reference for the further investigation and research of *H. rutilum* (Ker-Gawl.) Herb.

*Hippeastrum rutilum* (Ker-Gawl.) Herb. belongs to Amaryllidaceae, and *Hippeastrum* originated in Central and South America (Andradea et al. 2012). Its flower is large and brightly colored and its leaf form is beautiful, so there is high ornamental value in *Hippeastrum* (Wu et al. 2014). As a high-end ornamental flower, the relevant studies of *H*. *rutilum* mainly focused on cultivation technology, breeding, propagation technology, and pest control (Wang et al. 2020). The genetic background of the *Hippeastrum* is more complicated, and the genetic information for this species remains quite limited (Meerow et al. 2015). At present, there are few studies on the *H*. *rutilum* genome. The plastid genome is valuable in plant systematic research due to its highly conserved structures, uniparental inheritance, and haploid nature (Fu et al. 2016). The plastid genome has also been smartly engineered to confer useful agronomic traits and/or serve as bioreactors (Jin and Daniell 2015). Here, we have sequenced, assembled, and characterized the complete chloroplast genome sequence of *H. rutilum* (Ker-Gawl.) Herb. to provide information for the identification of *Hippeastrum*, as well as assist further phylogenetic studies of family Amaryllidaceae.

The sample used for chloroplast genome assembly is horticultural cultivation. The fresh leaves of *H. rutilum* (Ker-Gawl.) Herb. were sampled from Jinggangshan University (N27°06′45.46′′, E115°01′55.84′′), Ji’an, Jiangxi Province, China. The voucher specimen of *H. rutilum* has been kept in the Key Laboratory of Ecological Environment and Resource Utilization, Jinggangshan University. The specimen accession number is JGSU20190715. The total genomic DNA was extracted from fresh leaves using the modified CTAB method (Doyle and Doyle 1987). The DNA library was prepared with a TruSeq DNA Sample Prep Kit (Illumina, USA) according to the instructions of the manufacturer. Then the DNA library was sequenced on the Illumina Hiseq 2500 Sequencing System (Nanjing, China). Raw data were filtered to obtain high-quality clean data by removing adapter sequences and low-quality reads. 99.48% of raw data was obtained as high-quality clean data. The chloroplast genome was assembled via SPAdes v3.9.0 software (Bankevich et al. 2012). Annotation of the chloroplast genome was performed using the Dual Organellar GenoMe Annotator (DOGMA) online tool (Wyman et al. 2004), and Geneious v11.1.5 (Biomatters Ltd., Auckland, New Zealand) (Kearse et al. 2012) with MT133568.1 as the reference. Finally, we obtained a complete chloroplast genome of *H. rutilum* (Ker-Gawl.) Herb and submitted to GenBank with accession number (MT937175).

We found that another chloroplast genome of the same species was available in Genebank (*H. rutilum*) when we analyzed chloroplast genome characteristics of *H. rutilum* (Ker-Gawl.) Herb. The complete plastid genome sequence of *H. rutilum* (Ker-Gawl.) Herb. was 162,696 bp in length with 86,933 bp of the large single-copy (LSC) region, 5418 bp of the small single-copy (SSC) region, and 34,932 bp of the inverted repeats (IRs) regions. The complete cp genome of MT133568 was 158,357 bp in length, containing a large single-copy (LSC) region of 86,451 bp, a small single-copy (SSC) region of 18,272 bp, and two inverted repeat (IR) regions of 26,817 bp. The overall GC content of the plastid genome MT937175 was 37.68%, while the corresponding values of the LSC, SSC, and IR regions were 35.81, 31.67, and 40.48%, respectively. However, the overall GC content of the genome MT133568 was 37.93%, whereas the corresponding ratios of the LSC, SSC, and IR regions were 54.59, 11.54, and 33.87%, respectively. The genome MT937175 contained 128 genes, including 82 protein-coding genes, 38 tRNA genes, and 8 rRNA genes. The genome MT133568 has 133 genes in total, including 87 protein-coding genes, 38 tRNA genes, and 8 rRNA genes.

To investigate the phylogenetic relationship of *H. rutilum* (Ker-Gawl.) Herb., complete chloroplast genomes of 13 species were retrieved from Genebank. A molecular phylogenetic tree was constructed based on complete chloroplast genome sequences. The alignment was conducted using MAFFT v7.307 (Katoh and Standley 2013). The phylogenetic tree was built using RAxML (Stamatakis 2014) with a bootstrap set to 1000. The maximum likelihood(ML)phylogenetic analysis suggested *H. rutilum* (Ker-Gawl.) Herb. is closely related to *H*. *rutilum* (Figure 1). The results will not only help to investigate the population genetic diversity of *H*. *rutilum*, but also contribute to the molecular phylogeny studies of *Hippeastrum*.

**Figure 1.**
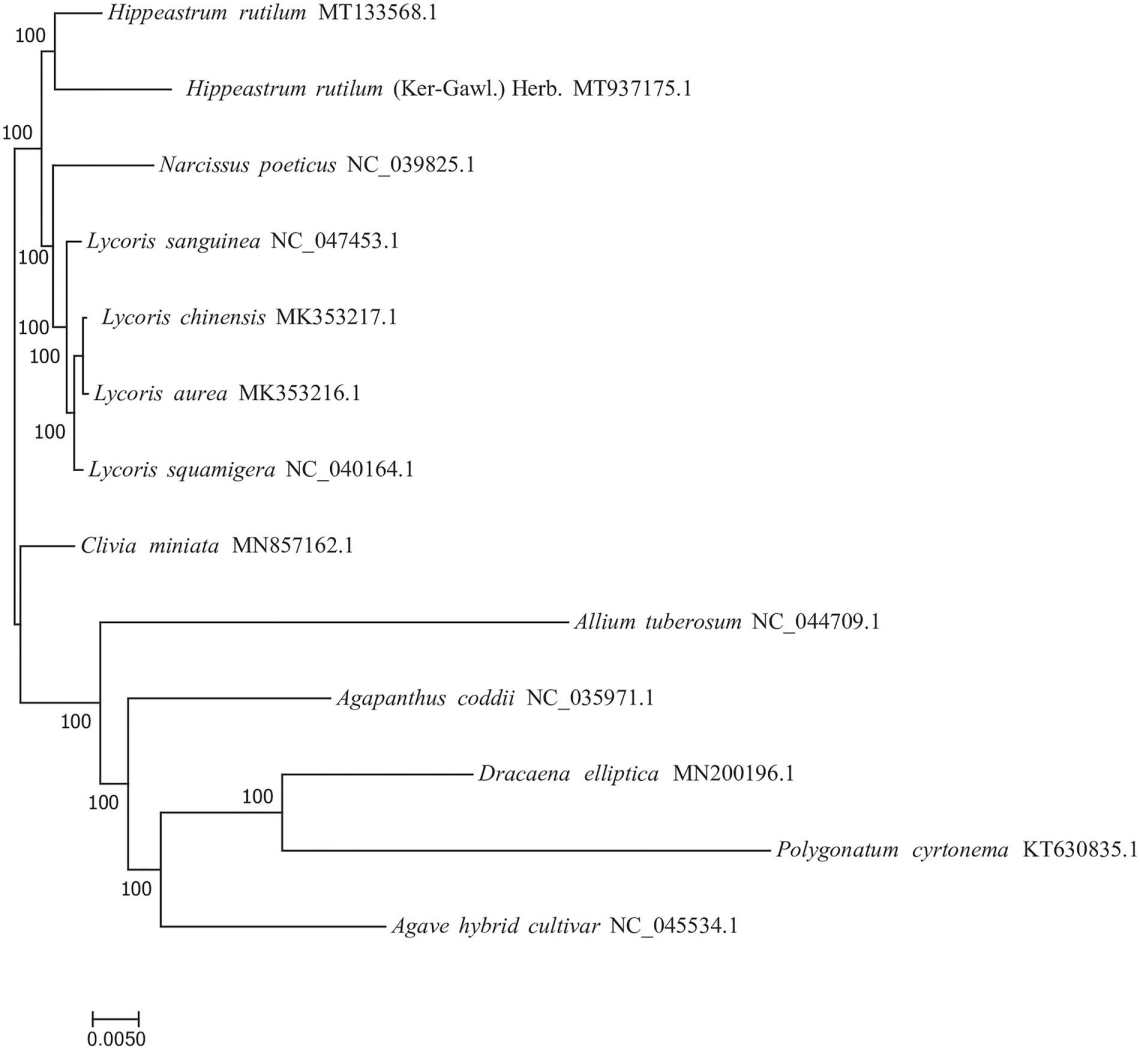
Phylogenetic tree inferred by maximum-likelihood (ML) method based on the complete chloroplast genome of 13 representative species. The bootstrap support values are shown at the branches.

## Data Availability

The genome sequence data that support the findings of this study are openly available in GenBank of NCBI at (https://www.ncbi.nlm.nih.gov/) under the accession no. MT937175. The associated BioProject, SRA, and Bio-Sample numbers are PRJNA668238, SRR12809558, and SAMN16401707 respectively.
